# Identification of Recurrence-Related microRNAs from Bone Marrow in Hepatocellular Carcinoma Patients

**DOI:** 10.3390/jcm4081600

**Published:** 2015-08-14

**Authors:** Keishi Sugimachi, Shotaro Sakimura, Akira Tomokuni, Ryutaro Uchi, Hidenari Hirata, Hisateru Komatsu, Yoshiaki Shinden, Tomohiro Iguchi, Hidetoshi Eguchi, Takaaki Masuda, Kazutoyo Morita, Ken Shirabe, Hidetoshi Eguchi, Yoshihiko Maehara, Masaki Mori, Koshi Mimori

**Affiliations:** 1 Department of Surgery, Kyushu University Beppu Hospital, 4546 Tsurumihara, Beppu 874-0838, Japan; E-Mails: sugimachi@beppu.kyushu-u.ac.jp (K.S.); z33sho2000@yahoo.co.jp (S.S.); ryuuchi@beppu.kyushu-u.ac.jp (R.U.); shusei_1300g7m7s@yahoo.co.jp (H.H.); komateru8312@gmail.com (H.K.); yshinden@beppu.kyushu-u.ac.jp (Y.S.); tomo@surg2.med.kyushu-u.ac.jp (T.I.); heguchi@beppu.kyushu-u.ac.jp (H.E.); takaakimas@yahoo.co.jp (T.M.); 2 Department of Surgery, Fukuoka City Hospital, 13-1 Yoshizukahonmachi, Fukuoka 812-0046, Japan; E-Mail: kamorita@surg2.med.kyushu-u.ac.jp; 3 Department of Gastroenterological Surgery, Graduate School of Medicine, Osaka University, 2-2 Yamadaoka, Suita, Osaka 565-0871, Japan; E-Mails: akiratomokuni@gmail.com (A.T.); heguchi@gesurg.med.osaka-u.ac.jp (H.E.); mmori@gesurg.med.osaka-u.ac.jp (M.M.); 4 Department of Surgery and Science Graduate School of Medical Sciences Kyushu University 3-1-1 Maidashi, Higashi-ku, Fukuoka 812-8582, Japan; E-Mails: kshirabe@surg2.med.kyushu-u.ac.jp (K.S.); maehara@surg2.med.kyushu-u.ac.jp (Y.M.)

**Keywords:** bone marrow, microRNA, hepatocellular carcinoma, recurrence

## Abstract

Hepatocellular carcinoma (HCC) is a poor-prognosis cancer due to its high rate of recurrence. microRNAs (miRNAs) are a class of small non-coding RNA molecules that affect crucial processes in cancer development. The objective of this study is to identify the role of miRNAs in patient bone marrow (BM) and explore the function of these molecules during HCC progression. We purified miRNAs from bone marrow cells of seven HCC patients, and divided them into three fractions by cell surface markers as follows: CD14^+^ (macrophage), CD14^−^/CD45^+^ (lymphocyte), and CD14^−^/CD45^−^/EpCAM^+^ (epithelial cell). We employed microarray-based profiling to analyze miRNA expression in the bone marrow of patients with HCC. Differentially expressed miRNAs were significantly different between fractions from whole bone marrow, macrophages, and lymphocytes, and depended on stages in tumor progression. Differences in expression of miRNAs associated with cell proliferation also varied significantly between HCC patients with recurrence, multiple tumors, and advanced clinical stages. These results suggest that miRNA profiles in separated fractions of BM cells are associated with HCC progression.

## 1. Introduction

Hepatocellular carcinoma (HCC) is the fourth most common malignancy in Japan and the fifth worldwide. The mainstay of its treatment is hepatic resection with improved outcomes; however, HCC is still characterized by frequent recurrence [1,2]. microRNAs (miRNAs) are small (17–21 nt), non-coding RNAs that regulate gene expression at the post-transcriptional level through the RNA interference pathway. Currently, ~2000 miRNAs have been described in humans, and a single miRNA may regulate many mRNAs. Through this mechanism, miRNAs are essential components in the regulation of many cellular and developmental processes, including developmental timing, organ development, differentiation, proliferation, immune regulation, and cancer development and progression [3]. Depending upon their target gene(s) and level of expression, miRNAs may function as either oncogenes or tumor suppressors and assist in the promotion or suppression of cancer growth and progression [4,5].

We previously demonstrated that circulating miRNAs in serum extracellular vesicles (exosomes) could be novel biomarkers for predicting the recurrence and therapeutic targets of HCC [6,7]. Exosomes are small membrane vesicles (30–100 nm) derived from the luminal membranes of multivesicular bodies and are constitutively released by fusion with the cell membrane [8]. Exosomes transfer not only membrane components but also nucleic acids to other cells; therefore, cell-derived exosomes have recently been described as a new mode of cell-to-cell communication [9]. To date, 764 microRNAs (miRs) have been identified in exosomes derived from several different cell types and from multiple organisms [10]. In this light, exosomally transported miRNAs have found a place in cancer research as carriers of genetic information [11,12]. Functional exosomal miRs both from cancer cells and bone marrow (BM) mesenchymal stem cells have been reported [13,14], but in a previous study we could not show direct evidence that exosomal serum miRNA was secreted from either HCC cells or other host cells, such as BM-derived cells [6]. Here we briefly report the miRNA profile of BM cells to understand the role of miRNAs in the progression of HCC.

## 2. Materials and Methods

Seven patients who consecutively underwent hepatectomy for primary HCC were selected from records of the Department of Gastroenterological Surgery, Osaka University. The institutional review board approved this study and we obtained written informed consent from each patient. The median age of the seven patients was 60 years (range 37–72); the etiologies were hepatitis C infection in four cases, hepatitis B in two, and alcoholism in one. The number of tumors was single in five cases and multiple in two cases. After the median follow-up time of 22 months, three cases had recurrence of HCC while four cases had no recurrence. On the basis of the UICC classification (7th edition), three cases were classified as stage I, two cases as stage II, and two cases as stage IIIA. 

Aspiration of BM was conducted under general anesthesia immediately before surgery, as previously described [15]. The BM aspirate was obtained from the sternum using a BM aspiration needle. A volume of 3 mL of BM was added to 4.0 mL of Isogen-LS (Nippon Gene, Toyama, Japan), which was shaken vigorously and stored at −80 °C until RNA extraction. BM cells were separated into three fractions using a three-step automagnetic-activated cell separation system (MACS) by MACS Cell Separators (Miltenyi Biotec, Bergisch Gladbach, Germany). CD45^+^, CD14^+^, and CD45^−^/EpCAM^+^ cell fractions were collected using CD45, CD14, and EpCAM (CD326) microbeads according to the manufacturer’s instructions (Miltenyi Biotec).

RNA was extracted from each BM fraction separated by the Auto MACS system using the miRNeasy Mini Kit (Qiagen, Venlo, The Netherlands) according to the manufacturer’s protocol. Extracted total RNA was labeled with Cy3 using the miRCURY LNA Array miR labeling kit (Exiqon, Vedbaek, Denmark). Labeled RNAs were hybridized onto 3D-Gene Human microRNA Oligo chips containing 837 anti-sense probes printed in duplicate spots (Toray, Kamakura, Japan). The annotation and oligonucleotide sequences of the probes conformed to the miRBase microRNA database (Faculty of Life Sciences, University of Manchester, Mancehster, UK). After stringent washes, fluorescent signals were scanned with the 3D-Gene Scanner (Toray) and analyzed using GenePix Pro version 5.0 (Molecular Devices, Sunnyvale, CA, USA). The raw data from each spot were normalized by subtraction of the background signal mean intensity, determined by the 95% confidence intervals of the signal intensities of all blank spots. Valid measurements were considered those in which the signal intensity of both duplicate spots was greater than two standard deviations of the background signal intensity. MicroRNAs differentially expressed between groups were statistically identified using the Welch *t*-test. Data was uploaded in Gene Expression Omnibus datasets (GSE71762, National Center for Biotechnology Information, Bethesda, MD, USA).

## 3. Results and Discussion

### 3.1. Whole BM Fraction

**Figure 1:**
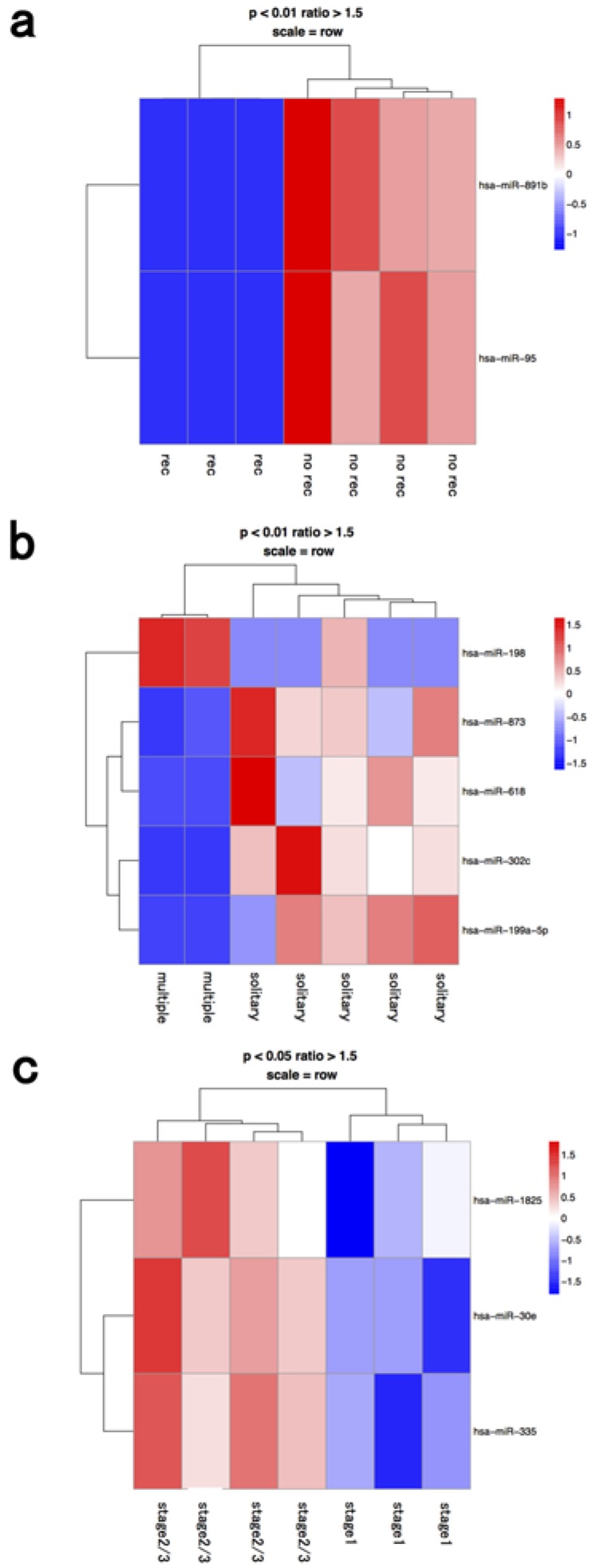
Hierarchical clustering analysis of microRNAs of whole bone marrow cells in seven patients with hepatocellular carcinoma. Heat map of the miRNA profile in bone marrow cells from hepatocellular carcinoma patients with (**a**) recurrence (*n* = 3) and no recurrence (*n* = 4); (**b**) solitary (*n* = 5) and multiple tumors (*n* = 2); (**c**) stage 1 (*n* = 3) and ≥stage 2 (*n* = 4). Cluster analysis showed two, five, and three miRNAs that were significantly differentially expressed between the two groups with a >1.50-fold change, respectively. Colors range from blue to red, corresponding to low to high expression, respectively. *p* values <0.01 or <0.05, unpaired *t* test. rec: recurrence.

miRNA profiles in the whole BM fraction are shown in Figure 1, depending on the recurrence factor, number of tumors, and clinical stage. Differentially expressed miRNAs were significantly different between fractions from whole bone marrow, macrophages, and lymphocytes (Table 1). Therefore, we further analyzed the miRNA profiles of macrophage and lymphocyte fractions. Our present data showed that miRNAs of selected fractions had to be independently analyzed with respect to the origin of the microRNAs present in the BM of HCC patients. miRNA processing may occur in the cancer cells themselves or in cells within the BM microenvironment, such as hematopoietic progenitor cells, endothelial cells, progenitor cells, and macrophages [16,17,18].

**Table 1:** MicroRNAs in the bone marrow cells that were significantly correlated with clinical significance of HCC patients.

BM Fraction	Clinical Significance	Upregulated microRNA	Downregulated microRNA
whole BM	recurrence		hsa-mir-891b
	recurrence		hsa-mir-95
	multiple	hsa-mir-198	hsa-mir-873
	multiple		hsa-mir-618
	multiple		hsa-mir-302c
	multiple		hsa-mir-199a-5p
	stage 2≥	hsa-mir-1825	
	stage 2≥	hsa-mir-30e	
	stage 2≥	hsa-mir-335	
lymphocyte	recurrence	hsa-mir-148a	hsa-mir-190
	recurrence	hsa-mir-361-5p	hsa-mir-503
	recurrence	hsa-mir-320d	hsa-mir-544
	multiple	hsa-mir-654-5p	hsa-mir-517a
	multiple		hsa-mir-497
	multiple		hsa-mir-454
	multiple		hsa-mir-22
	stage 2≥	hsa-mir-1537	hsa-mir-345
	stage 2≥	hsa-mir-513b	hsa-mir-553
	stage 2≥	hsa-mir-15a	hsa-mir-653
	stage 2≥	hsa-mir-517a	hsa-mir-577
	stage 2≥	hsa-mir-28-5p	
macrophage	recurrence	hsa-mir-1207-3p	hsa-mir-1277
	recurrence	hsa-mir-937	hsa-mir-1279
	recurrence		hsa-mir-184
	recurrence		hsa-mir-563
	recurrence		hsa-mir-96
	recurrence		hsa-mir-302b
	multiple	hsa-mir-1	hsa-mir-10b
	multiple	hsa-mir-889	hsa-mir-204
	multiple	hsa-mir-658	hsa-mir-654-3p
	multiple		hsa-mir-302a
	stage 2≥	hsa-mir-555	hsa-mir-942
	stage 2≥	hsa-mir-1293	hsa-mir-1227
	stage 2≥		hsa-mir-598
	stage 2≥		hsa-mir-518d-3p

BM: bone marrow.

### 3.2. Lymphocyte Fraction

**Figure 2:**
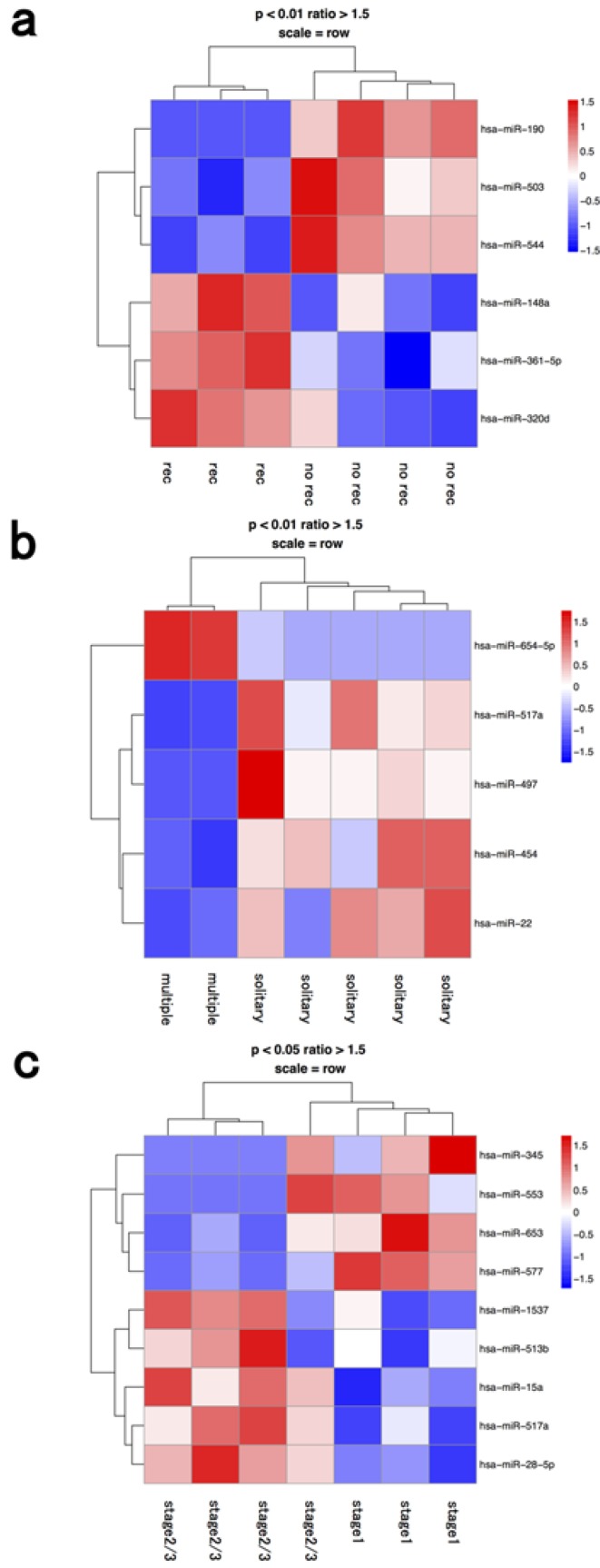
Hierarchical clustering analysis of microRNAs of lymphocyte fraction in seven patients with hepatocellular carcinoma. Heat map of the miRNA profile in macrophages from hepatocellular carcinoma patients with (**a**) recurrence (*n* = 3) and no recurrence (*n* = 4); (**b**) solitary (*n* = 5) and multiple tumors (*n* = 2); (**c**) stage 1 (*n* = 3) and ≥stage 2 (*n* = 4). Cluster analysis showed six, five, and nine miRNAs that were significantly differentially expressed between the two groups with a >1.50-fold change, respectively. Colors range from blue to red, corresponding to low to high expression, respectively. *p* values <0.01 or <0.05, unpaired *t* test. rec: recurrence.

Inflammation appears to be a crucial factor in hepatocarcinogenesis since HCC typically occurs in patients with chronic inflammatory liver diseases, such as viral hepatitis or non-alcoholic steatohepatitis [19,20]. One miR (hsa-miR-654-5p) was upregulated and four miRs (hsa-miR-517a, 497, 454, 22) were downregulated in the lymphocyte fraction of cases with multiple tumors compared to ones with a solitary tumor (Figure 2 and Table 1). miR-517a is located in the chromosome 19 miRNA cluster, and is considered to be a tumor-suppressive miRNA that is suppressed by epigenetic modifications [21]. miR-517a inhibited cell proliferation by blocking G2/M transition in HCC [21], and markedly induced bladder cancer cell apoptosis [22]. One of the target genes of miR-517a in HCC was reported to be Pyk2, which was associated with MAP kinase signaling pathways [23]. miR-497, clustered at 17p13.1, is also reported to be a tumor suppressor and shows significant growth-suppressive activity with induction of G1 arrest in HCC [24]. Potential target genes of miR-497 in cancers had been reported to be insulin-like growth factor 1 receptor, WEE1, HDGF, VEGF-A, Akt, and IKKβ [25,26,27,28].

### 3.3. Macrophage Fraction

**Figure 3:**
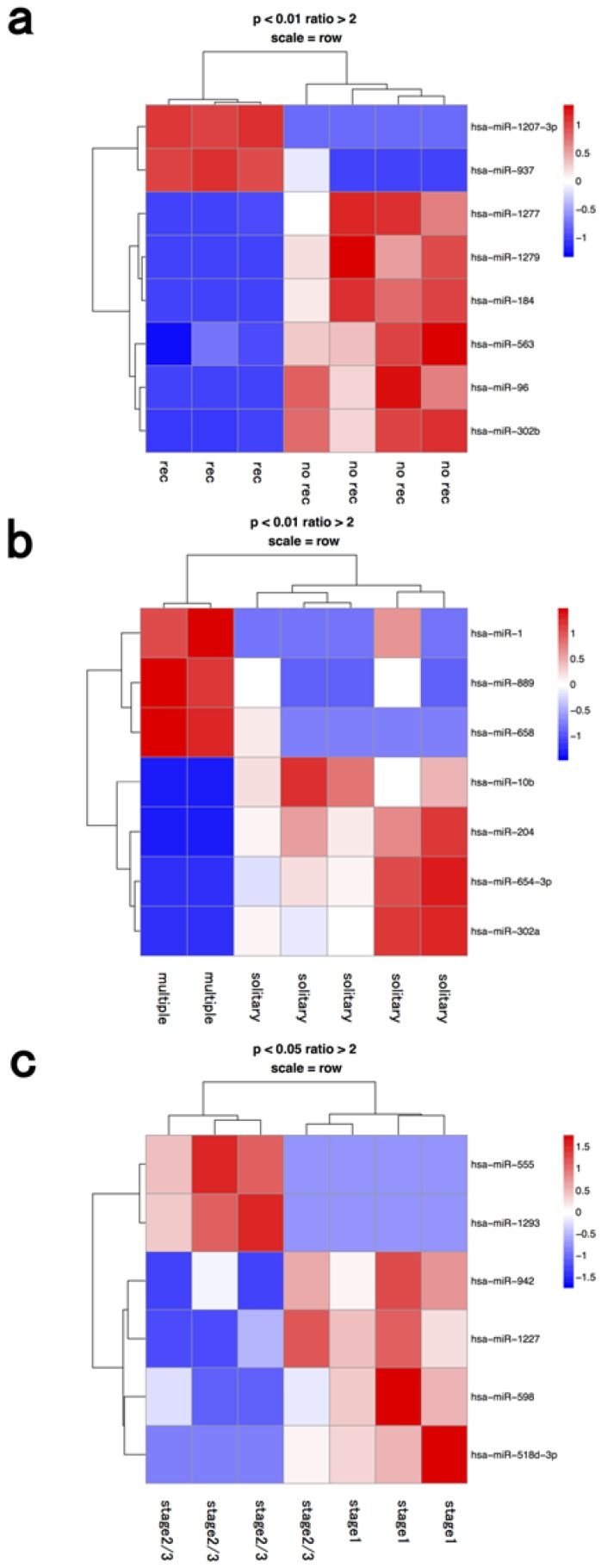
Hierarchical clustering analysis of microRNAs of macrophage fraction in seven patients with hepatocellular carcinoma. Heat map of the miRNA profile in lymphocytes from hepatocellular carcinoma patients with (**a**) recurrence (*n* = 3) and no recurrence (*n* = 4); (**b**) solitary (*n* = 5) and multiple tumors (*n* = 2); (**c**) stage 1 (*n* = 3) and ≥stage 2 (*n* = 4). Cluster analysis showed eight, seven, and six miRNAs that were significantly differentially expressed between the two groups with a >2-fold change, respectively. Colors range from blue to red, corresponding to low to high expression, respectively. *p* values <0.01 or <0.05, unpaired *t* test. rec: recurrence.

Two miRNAs were upregulated (hsa-miR-1207-3p, 937) and six miRNAs (hsa-miR-1277, 1279, 184, 563, 96, 302b) were downregulated in the macrophage fractions from cases with post-operative recurrence compared to fractions from cases with no recurrence (Figure 3 and Table 1). 

Previous studies have shown that miR-184 can act either as an oncogenic- or a tumor suppressive-miRNA in various human cancers, depending on cellular context [29,30]. Lin *et al.* reported that decreased miR-184 promotes cancer cell invasiveness by an increase in CDC25A and c-myc expression [31]. In HCC, miR-302b acts as a tumor suppressor by targeting AKT2, suppressing G1 regulators (Cyclin A, Cyclin D1, CDK2) and increasing p27Kip1 phosphorylation at Ser10 [32]. Recent studies have revealed that tumor-associated macrophages (TAMs) are an important component of the tumor microenvironment and can promote tumor progression [35,36]. TAMs were reported to be associated with metastasis, angiogenesis, epithelial-mesenchymal transition (EMT), and poor prognosis in HCC [35,37,38]. Macrophages have multiple biological roles, including antigen presentation, target cell cytotoxicity, removal of foreign bodies, tissue remodeling, regulation of inflammation, induction of immunity, thrombosis, and endocytosis. Aucher *et al.* recently reported that transfer of miRNAs from macrophages functionally inhibited proliferation of HCC cells [39]. Although the mechanisms by which TAMs promote tumor progression are poorly understood, our data implied that they might act through altered miRNA expression.

## 4. Conclusions

These results suggest that miRNA profiles in separated fractions of bone marrow cells are associated with metastasis, angiogenesis, epithelial-mesenchymal transition (EMT), and poor prognosis in HCC. In this study, the number of cases was small, therefore our data are preliminary and further analysis including validation studies with large cohorts and *in vitro* studies to search target genes and pathways of identified miRNAs are warranted. We revealed the miR profiling of BM cells associated with stage/recurrence of HCC in this study. It is possible that miR profiling of BM niche was affected by the progression of HCC or disseminated cancer cells. On the other hand, altered miR expression of BM cells might help the survival or proliferation of cancer cells in BM. At present, it is difficult to conclude that one specific mechanism is superior to others, and further studies are recommended. Our data could provide a database to seek new concepts in immunotherapy targeting miRNAs of BM cells to improve the outcome of patients with HCC in the near future.
